# Simultaneous Determination of Four Active Ingredients in* Sargentodoxa cuneata* by HPLC Coupled with Evaporative Light Scattering Detection

**DOI:** 10.1155/2016/8509858

**Published:** 2016-05-29

**Authors:** Di-Hua Li, Yuan-Shan Lv, Jun-Hong Liu, Lei Yang, Yan Wang, Shu-Kun Zhang, Yu-Zhen Zhuo

**Affiliations:** ^1^Tianjin Institute of Acute Abdominal Diseases of Integrated Traditional Chinese and Western Medicine, Tianjin 300100, China; ^2^College of Pharmacy, Tianjin University of Traditional Chinese Medicine, Tianjin 300193, China

## Abstract

A HPLC coupled with evaporative light scattering detection method had been developed for the simultaneous determination of 3,4-dihydroxyphenylethyl alcohol glycoside, salidroside, chlorogenic acid, and liriodendrin in the stem of* Sargentodoxa cuneata*. With a C18 column, the analysis was performed using acetonitrile and 0.2% formic acid aqueous solution as mobile phase in gradient program at a flow rate of 0.9 mL/min. The optimum drift tube temperature of evaporative light scattering detection was at 105°C with the air flow rate of 2.5 L/min. The calibration curves showed good linearity during the test ranges. This method was validated for limits of detection and quantification, precision, and reproducibility. The recoveries were within the range of 96.39%–104.64%. The relative standard deviations of intraday and interday precision were less than 2.90% and 3.30%, respectively. The developed method can be successfully used to quantify the four analytes in the stem of* Sargentodoxa cuneata* from various regions in China.

## 1. Introduction

The stem of* Sargentodoxa cuneata* (Oliv.) Rehd. et Wils (Sargentodoxaceae) is a well-known herb of traditional Chinese medicine in China for treating abdominal pain due to acute appendicitis, sore and ulcer, dysmenorrheal, amenorrhea, traumatic swelling, and rheumatic arthritis [1]. According to reports in the literature, it possesses various pharmacological effects such as antiviral [2, 3], antibacterial [4], antioxidant [5, 6], antitumor [7], anti-inflammatory [8], antimyocardial ischemia [9], and antithrombotic [10].

Phytochemical investigations have showed that there mainly are organic acids, phenolic glycosides, lignins, triterpene saponins, polysaccharides, and other compounds in the stem of* S. cuneata*. Among these chemical components, the main active ingredients are believed to be salidroside, chlorogenic acid, and liriodendrin. These compounds are reported to possess effects such as antitumor [11], antiaging [12], antifatigue [13], hypolipidemic [14], antiviral [15], antiallergy [16], antioxidant [17], anti-inflammatory, analgesic [18], antimyocardial ischemia [9], antiarrhythmic [19], and calcium channel antagonistic [20]. Another phenolic glycoside such as 3,4-dihydroxyphenylethyl alcohol glycoside has also been isolated from the stem of* S. cuneata* in recent years [21]. Although the pharmacological effect of 3,4-dihydroxyphenylethyl alcohol glycoside has not been reported, its aglycone (hydroxytyrosol) has obvious pharmacological effects such as antioxidant [22], anti-inflammatory [23], and antitumor [24, 25]. So 3,4-dihydroxyphenylethyl alcohol glycoside can be temporarily deemed to be a potentially biological active component.

In order to evaluate the quality of the stem of* S. cuneata*, HPLC with UV detection methods have been reported previously for measurement of chlorogenic acid [26], salidroside [27] or emodin [28], and so forth in the stem of* S. cuneata*. Therefore, it is necessary to develop a simple, economical, and efficient method analyzing multiple biological active components in the stem of* S. cuneata*. However, for liriodendrin having only a weak UV absorption at maximum absorption wavelength (271 nm) and the components with different maximum absorption wavelength, the use of UV detector was limited for simultaneous analyzing of all these components. And the interference of end absorption of ultraviolet is serious for analysis of* S. cuneata*. Therefore, it is reported in this paper that applying evaporative light scattering detection (ELSD) can resolve these problems. Because ELSD response does not depend on the optical characteristics of the sample, it will meet the need of simultaneous determination of components with different maximum UV absorption and can improve the detection sensitivity of components with weak UV absorption.

In the present study, an HPLC-ELSD method has been developed to simultaneously determine 3,4-dihydroxyphenylethyl alcohol glycoside, salidroside, chlorogenic acid, and liriodendrin in the stem of* S. cuneata* from various regions in China for the first time. Their contents in different samples of* S. cuneata* have also been compared.

## 2. Materials and Methods

### 2.1. Chemicals and Reagents

**Figure 1:**
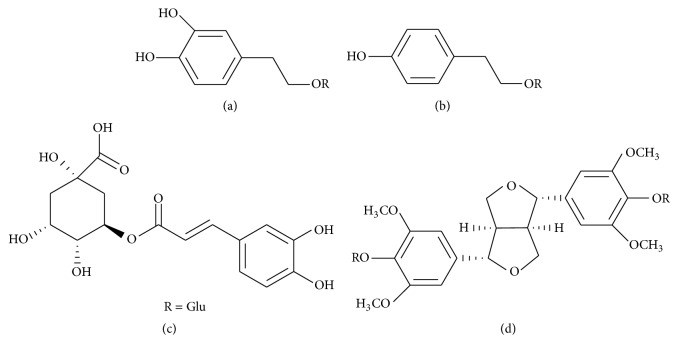
Chemical structure of the standard compounds in the present study. (a) 3,4-Dihydroxyphenylethyl alcohol glycoside; (b) salidroside; (c) chlorogenic acid; (d) liriodendrin.

The standards of salidroside and chlorogenic acid were purchased from the Chinese National Institute of Control of Pharmaceutical and Biological Products (Beijing, China). The standards of 3,4-dihydroxyphenylethyl alcohol glycoside and liriodendrin [29] were isolated by the author from the stem of* S. cuneata* using preparative high performance liquid chromatography and resin. And their structures were characterized by spectroscopic data analysis (^1^H-NMR, ^13^C-NMR). Their purities were determined to be over 98% by normalization of the peak areas by HPLC. The structures of the four compounds are provided in Figure 1. HPLC-grade acetonitrile, methanol, and formic acid were bought from Concord Technology Co., Ltd. (Tianjin, China). Water was ultrapure water.

### 2.2. Plant Materials

Samples of the stem of* S. cuneata* were obtained from various regions in China. All samples were confirmed in accordance with Pharmacopoeia of the People's Republic of China and the voucher specimens were deposited at Institute of Acute Abdominal Diseases of Integrated Traditional Chinese and Western Medicine, Tianjin, China.

### 2.3. Apparatus and HPLC-ELSD Separation Procedure

The HPLC system consisted of a SSI PC2000 chromatograph, an ultraviolet (UV) Model 500 detector (SSI, scientific Systems. Inc., USA), an ELSD 2000ES detector (Alltech, Alltech Associates. Inc., USA), and a column oven. A Hypersil ODS (C18) column (250 mm × 4.6 mm id, 5 *μ*m) was operated at 35°C. Solvents that constituted the mobile phase were acetonitrile (A) and 0.2% formic acid aqueous solution (B). The separation was performed using a stepwise gradient elution of 0–14 min, 95–87% B; 14–25 min, 87-87% B; 25–45 min, 87–81% B, then keeping 95% B to balance for 10 min. The flow rate was 0.9 mL/min. ELSD was used as the detection method with nebulizing gas flow rate of 2.5 L/min and drift tube temperature of 105°C. The impactor position of ELSD was set off.

### 2.4. Preparation of Sample Solutions

The stem of* S. cuneata* was powdered to a homogeneous size in a mill, sieved through a No. 65 mesh. The powder of sample (0.5 g) was placed into a 50 mL glass round flask and accurate 25 mL 60% methanol was added. Then the flask was accurately weighed and extracted for 30 min in ultrasonic bath (power 250 W, frequency 33 KHz), allowed to cool (60% methanol was used to compensate the loss of the solvent), and mixed well. The successive filtrates of all samples were filtered through 0.45 *μ*m Millipore filter before that 20 *μ*L of the solution was injected into the HPLC system.

### 2.5. Methods Validation

#### 2.5.1. Calibration Curves

The 60% methanol stock solution containing 3,4-dihydroxyphenylethyl alcohol glycoside, salidroside, chlorogenic acid, and liriodendrin was prepared and diluted in seven concentrations of 21.6–288, 15.6–208, 16.05–214, and 15.45–206 *μ*g/mL, respectively. The calibration curve graphs were constructed by plotting the logarithm peak area versus the logarithm mass of each analyte [30].

#### 2.5.2. LOD and LOQ

The stock solution was diluted in proper concentration with 60% methanol. Then the diluted solution was injected into HPLC system for analysis. The LOD and LOQ under the present chromatographic conditions were determined at S/N about 3 and 10, respectively.

#### 2.5.3. Precision and Reproducibility

In order to check the precision of this method, the mixed standard solution was analyzed by HPLC-ELSD as mentioned above. The intraday precision was tested six times and the interday precision was tested on 6 days. The precision was expressed by calculating the relative standard deviations (RSD) of the four analytes, respectively.

To confirm the reproducibility, one sample from Sichuan province was extracted independently six times and analyzed by HPLC-ELSD as mentioned above. The RSD of the four analytes was used as the measurement of reproducibility.

#### 2.5.4. Recovery

Recovery test was performed to evaluate the accuracy as well as the extraction recovery. To determine the recovery, the contents of the four analytes in a sample were estimated according to their respective calibration curve. Different amount of standard compounds was mixed with a certain sample at ca. 0.8, 1.0, and 1.2 times of the estimated mass of each analyte and then extracted and analyzed using the method described above. The experiments were repeated three times for each level. For comparison, the blank sample (sample of* S. cuneata* without spiking four standard compounds) was also extracted and analyzed using the method described above. Extraction recoveries were calculated as follows: Recovery (%) = 100 × (amount determined − original amount)/amount spiked.

## 3. Results

### 3.1. Method Validation

#### 3.1.1. Linearity, LOD, and LOQ

**Figure 2:**
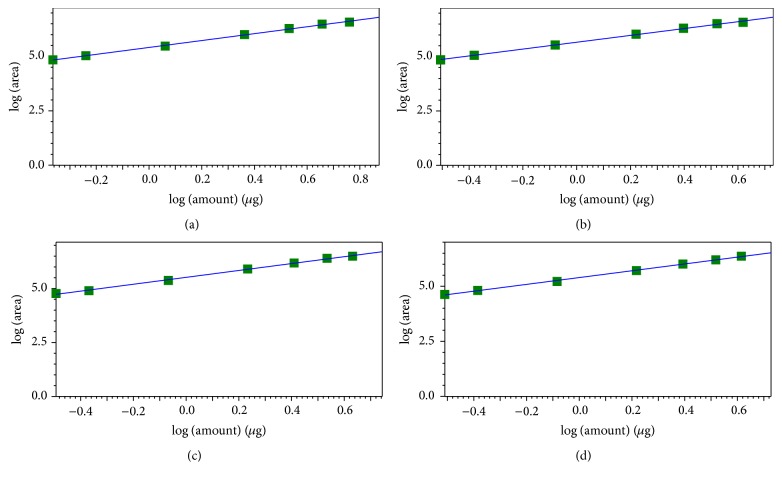
The calibration curves for the four analytes in the present study. (a) 3,4-Dihydroxyphenylethyl alcohol glycoside; (b) salidroside; (c) chlorogenic acid; (d); liriodendrin.

The calibration curve graphs of 3,4-dihydroxyphenylethyl alcohol glycoside, salidroside, chlorogenic acid, and liriodendrin were obtained using the developed HPLC-ELSD method and were shown in Figure 2. All the calibration curves showed good linearity. The ranges of LOD and LOQ for the four analytes under the present chromatographic conditions were 0.0296–0.1126 *μ*g and 0.1184–0.2695 *μ*g. The detailed results were shown in Table 1.

#### 3.1.2. Precision and Reproducibility

The precision and reproducibility of this method were good for quantifying the stem of* S. cuneata*. The RSDs of the intraday and interday for a 6-d period were less than 2.90% and 3.30%, respectively. The RSDs of retention time and peak area for the four analytes in the investigation of reproducibility were less than 0.57% and 2.11%, respectively. The reproducibility of the four analytes was consistent.

#### 3.1.3. Recovery

A sample (Sichuan province) was extracted and analyzed independently three times using the method described above and the contents of 3,4-dihydroxyphenylethyl alcohol glycoside, salidroside, chlorogenic acid, and liriodendrin were calculated to be 5.187, 4.155, 5.393, and 3.544 mg/g, respectively, according to their calibration curves.

A known amount of each standard compound at three different levels (1.04, 1.30, and 1.56 mg of 3,4-dihydroxyphenylethyl alcohol glycoside, 0.80, 1.00, and 1.20 mg of salidroside, 1.08, 1.35, and 1.62 mg of chlorogenic acid, and 0.68, 0.85, and 1.02 mg of liriodendrin) was mixed with 0.25 g of the sample (Sichuan province). These samples were processed and measured as described above and the recoveries for the four analytes were calculated. The results of recoveries were shown in Table 2. The recoveries ranged from 96.39% to 104.64% and were satisfactory.

### 3.2. Sample Analysis

**Figure 3:**
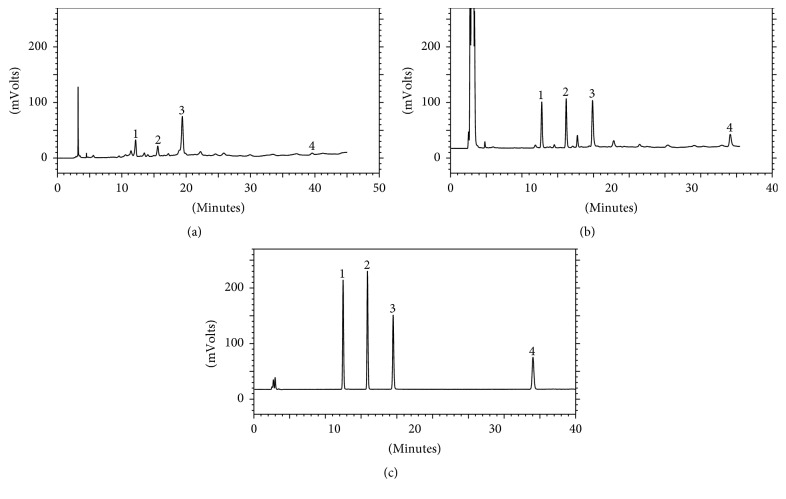
HPLC chromatograms diagrams. Sample at 271 nm (a); sample with ELSD (b); standard solution with ELSD (c). Peak: (1) 3,4-dihydroxyphenylethyl alcohol glycoside; (2) salidroside; (3) chlorogenic acid; (4) liriodendrin.

The developed HPLC-ELSD method was applied to simultaneous determination of 3,4-dihydroxyphenylethyl alcohol glycoside, salidroside, chlorogenic acid, and liriodendrin in 13 test samples and the contents of the analytes were revealed in Table 3. The typical chromatograms for the four standard compounds and for a sample were shown in Figure 3. As shown in Table 3, the contents of the four analytes ranged from 1.277 ± 0.042 to 10.356 ± 0.008 mg/g for 3,4-dihydroxyphenylethyl alcohol glycoside, 0.795 ± 0.028 to 4.411 ± 0.194 mg/g for salidroside, 3.017 ± 0.014 to 7.671 ± 0.247 mg/g for chlorogenic acid, and 1.809 ± 0.119 to 3.185 ± 0.067 mg/g for liriodendrin, respectively. Liriodendrin had the least content variation range among the four analytes.

## 4. Discussion

### 4.1. Optimization of Preparation of Sample Solutions

According to the solubility of the four investigated compounds, the paper optimized the concentration of extraction solvent such as methanol, 80% methanol, 60% methanol, and 40% methanol solution. At the same time, extraction time and solvent volume of processing procedure were also optimized by the developed method. The results indicated that the best extraction process was using 50 times 60% methanol for 30 min.

### 4.2. Optimization of Chromatograph Condition

In the experiment, the four investigated compounds could not be separated effectively by using the isocratic mobile solvents. After this separation was performed using a gradient system, which was composed of different combination of acetonitrile and 0.2% formic acid aqueous solution, the paper obtained a satisfactory chromatogram. Moreover, the different kind of acids (formic acid, acetic acid glacial, etc.) was also investigated. The stable chromatograph can be obtained by using 0.2% formic acid aqueous solution.

For components having only weak UV absorption at maximum absorption wavelength, such as liriodendrin, and different maximum absorption wavelength, though UV detector could determine them, the detection sensitivity was limited. Through UV chromatogram compared with ELSD chromatogram, the paper found that ELSD chromatogram of the four analytes was better than UV chromatogram and ELSD could improve the detection sensitivity. Chromatogram comparisons of them were shown in Figure 3.

### 4.3. Analysis of the Content Changes

The contents of the investigated compounds in 13 test samples were different considerably. The data of the study was consistent with the reports in the literature [31], probably because they were derived from the plant origins, environmental factors, and some other factors, such as harvesting season, drying process, and storage conditions. In the future, we plan to use this HPLC-ELSD method and chemical fingerprint [32] to analyze samples with various factors, so that the changes of content were understood further.

## 5. Conclusion

A simple HPLC-ELSD method for the simultaneous determination of 3,4-dihydroxyphenylethyl alcohol glycoside, salidroside, chlorogenic acid, and liriodendrin in the stem of* S. cuneata* has been developed for the first time. The method was rapid, linear, accurate, and reproducible. The results of 13 test samples in Table 3 have suggested that the method can be used as a quality control method of* S. cuneata* (for both qualitative and quantitative analysis).

## Figures and Tables

**Table 1:** Linear equations, linear range, LOD, and LOQ for analytes.

Analytes	Retention time (min)^a^	Linear equations^b^	*r* ^c^	Linear range (*μ*g)	LOD (*μ*g)	LOQ (*μ*g)
3,4-Dihydroxyphenylethyl alcohol glycoside	12.445 ± 0.098	*y* = 1.58697*x* + 5.41159	0.9992	0.432–5.76	0.0712	0.2136
Salidroside	15.820 ± 0.095	*y* = 1.57417*x* + 5.66256	0.9991	0.312–4.16	0.0296	0.1184
Chlorogenic acid	19.483 ± 0.092	*y* = 1.59715*x* + 5.51731	0.9991	0.321–4.28	0.1126	0.1689
Liriodendrin	38.920 ± 0.169	*y* = 1.54647*x* + 5.39389	0.9993	0.309–4.12	0.1030	0.2695

^a^Mean ± SD

^b^ *y* and *x* denote the logarithm value of peak area and mass, respectively.

^c^Correlation coefficient.

**Table 2:** Recoveries of spiked analytes.

Analytes	Original (mg)	Spiked (mg)	Found (mg)	Recovery (%)	Mean (%)	RSD (%)
3,4-Dihydroxyphenylethyl alcohol glycoside	1.297		1.875 ± 0.0078	100.59 ± 0.93		
	1.302	1.040	1.905 ± 0.0049	103.71 ± 0.59	98.12	2.38
	1.296		1.898 ± 0.0191	103.45 ± 2.29		
	1.299		2.078 ± 0.0035	99.84 ± 0.34		
	1.292	1.300	2.049 ± 0.0198	97.63 ± 1.90	102.58	1.69
	1.294		2.017 ± 0.0071	94.40 ± 0.68		
	1.310		2.336 ± 0.0057	100.35 ± 0.45		
	1.320	1.560	2.337 ± 0.0057	101.15 ± 0.45	102.55	0.69
	1.297		2.308 ± 0.0184	104.26 ± 1.47		

Salidroside	1.039		1.494 ± 0.0057	102.21 ± 0.97		
	1.043	0.800	1.492 ± 0.0035	100.00 ± 1.41	103.62	0.94
	1.038		1.500 ± 0.0085	96.30 ± 0.71		
	1.041		1.651 ± 0.0078	103.56 ± 0.88		
	1.035	1.000	1.628 ± 0.0113	102.67 ± 0.55	99.50	3.00
	1.037		1.600 ± 0.0057	104.63 ± 1.33		
	1.049		1.855 ± 0.0134	105.76 ± 1.40		
	1.057	1.200	1.823 ± 0.0141	101.81 ± 1.47	104.64	2.35
	1.039		1.852 ± 0.0014	106.33 ± 0.15		

Chlorogenic acid	1.348		1.985 ± 0.0212	104.93 ± 2.46		
	1.354	1.080	1.997 ± 0.0198	105.76 ± 2.29	103.64	2.88
	1.347		1.944 ± 0.0078	100.22 ± 0.90		
	1.351		2.179 ± 0.0021	101.64 ± 0.20		
	1.343	1.350	2.133 ± 0.0064	97.97 ± 0.59	100.68	2.36
	1.346		2.183 ± 0.0057	102.43 ± 0.52		
	1.362		2.469 ± 0.0099	106.44 ± 0.76		
	1.373	1.620	2.438 ± 0.0163	103.33 ± 1.25	104.36	1.72
	1.348		2.418 ± 0.0233	103.33 ± 1.80		

Liriodendrin	0.886		1.255 ± 0.0071	100.40 ± 1.30		
	0.890	0.680	1.263 ± 0.0028	101.35 ± 0.52	101.61	1.34
	0.885		1.269 ± 0.0057	103.08 ± 1.04		
	0.888		1.363 ± 0.0007	95.92 ± 0.10		
	0.882	0.850	1.368 ± 0.0035	97.28 ± 0.52	96.39	0.80
	0.884		1.360 ± 0.0042	95.97 ± 0.62		
	0.895		1.539 ± 0.0007	100.81 ± 0.09		
	0.902	1.020	1.531 ± 0.0085	99.20 ± 1.04	99.23	1.57
	0.886		1.506 ± 0.0057	97.70 ± 0.69		

**Table 3:** The contents of the four analytes in the stem of *S. cuneata* (*n* = 3, mean ± SD, mg/g).

Number	Source	3,4-Dihydroxyphenylethyl alcohol glycoside	Salidroside	Chlorogenic acid	Liriodendrin
1	Hunan province	10.356 ± 0.008	3.146 ± 0.071	4.874 ± 0.180	2.207 ± 0.052
2	Anhui province	5.969 ± 0.097	2.938 ± 0.135	3.961 ± 0.042	2.659 ± 0.040
3	Guizhou province	3.718 ± 0.151	3.042 ± 0.118	5.112 ± 0.178	2.209 ± 0.136
4	Yunnan province	6.753 ± 0.201	3.847 ± 0.114	5.147 ± 0.114	2.654 ± 0.005
5	Guangxi autonomous region	7.865 ± 0.184	3.105 ± 0.036	6.083 ± 0.095	2.201 ± 0.051
6	Henan province	6.660 ± 0.025	2.458 ± 0.020	5.789 ± 0.331	2.092 ± 0.070
7	Guangdong province	5.769 ± 0.013	2.549 ± 0.091	6.156 ± 0.038	2.124 ± 0.023
8	Jiangsu province	8.296 ± 0.164	4.411 ± 0.194	5.210 ± 0.115	2.488 ± 0.128
9	Hubei province	1.277 ± 0.042	0.989 ± 0.041	3.017 ± 0.014	1.809 ± 0.119
10	Jiangxi province	7.607 ± 0.349	2.731 ± 0.114	6.151 ± 0.179	2.135 ± 0.201
11	Zhejiang province	1.733 ± 0.007	1.783 ± 0.064	3.517 ± 0.022	2.487 ± 0.077
12	Hainan province	1.717 ± 0.064	0.795 ± 0.028	7.671 ± 0.247	2.368 ± 0.007
13	Sichuan province	5.187 ± 0.099	4.155 ± 0.048	5.393 ± 0.185	3.544 ± 0.019
